# Neovaginal Diversion Colitis Successfully Treated With Mesalazine Suppositories: Endoscopic Documentation of Healing

**DOI:** 10.1111/den.70100

**Published:** 2026-01-14

**Authors:** Shinichiro Kawatoko, Marimo Mori, Junji Umeno

**Affiliations:** ^1^ Department of Medicine and Clinical Science, Graduate School of Medical Sciences Kyushu University Fukuoka Japan; ^2^ Department of Internal Medicine Kyushu University Beppu Hospital Oita Japan

**Keywords:** mesalazine, neovaginal diversion colitis, vaginoscopy

A 41‐year‐old woman with Mayer–Rokitansky–Küster–Hauser syndrome (type I), a congenital absence of the uterus and upper vagina, underwent sigmoid colon vaginoplasty at age 18 and was followed at our gynecology department. She presented with a 10‐month history of intermittent neovaginal bleeding and dyspareunia. A manual neovaginal examination was unremarkable, but persistent symptoms prompted referral to our department (Gastroenterology) for further evaluation. Vaginoscopy (EVIS X1 and GIF‐XZ1200, Olympus Corp., Tokyo, Japan) revealed continuous erythema, edema, erosions, and loss of vascular pattern, resembling ulcerative colitis, in the neovaginal sigmoid colon (Figure 1a,b). Biopsy histology showed chronic active colitis with crypt distortion, cryptitis, and goblet cell depletion (Figure 1c,d). Bacterial culture and *Treponema pallidum* PCR were negative. Colonoscopy showed only minor aphthous erosions confined to the anal verge, with no lesions in the upstream rectum or colon. She had no gastrointestinal symptoms suggestive of ulcerative colitis, such as diarrhea, hematochezia, or abdominal pain. From these findings, she was diagnosed with isolated neovaginal diversion colitis. She was initially treated with intravaginal budesonide foam enemas (2 mg, once daily) for 7 weeks and advised to refrain from vaginal intercourse; however, symptoms persisted. Subsequently, mesalazine vaginal suppositories (1000 mg, once daily) were initiated with the aid of a vaginal prosthesis to facilitate drug delivery to the neovaginal blind end, resulting in marked symptom improvement within 1 week. Follow‐up vaginoscopy after 7 weeks demonstrated mucosal normalization, and histology confirmed resolution of inflammation (Figure 2). She resumed vaginal intercourse while continuing mesalazine suppositories, with only occasional trace bleeding and no other symptoms for 13 months. Neovaginal diversion colitis, also called “diversion neovaginitis,” may result from nutritional deprivation (e.g., short‐chain fatty acids) [1]. Despite treatments like short‐chain fatty acid enemas, mesalazine, or corticosteroids, no standardized treatment has been established [1, 2]. Accurate endoscopic and histological assessment enables effective medical management and preserves sexual function.

**FIGURE 1:**
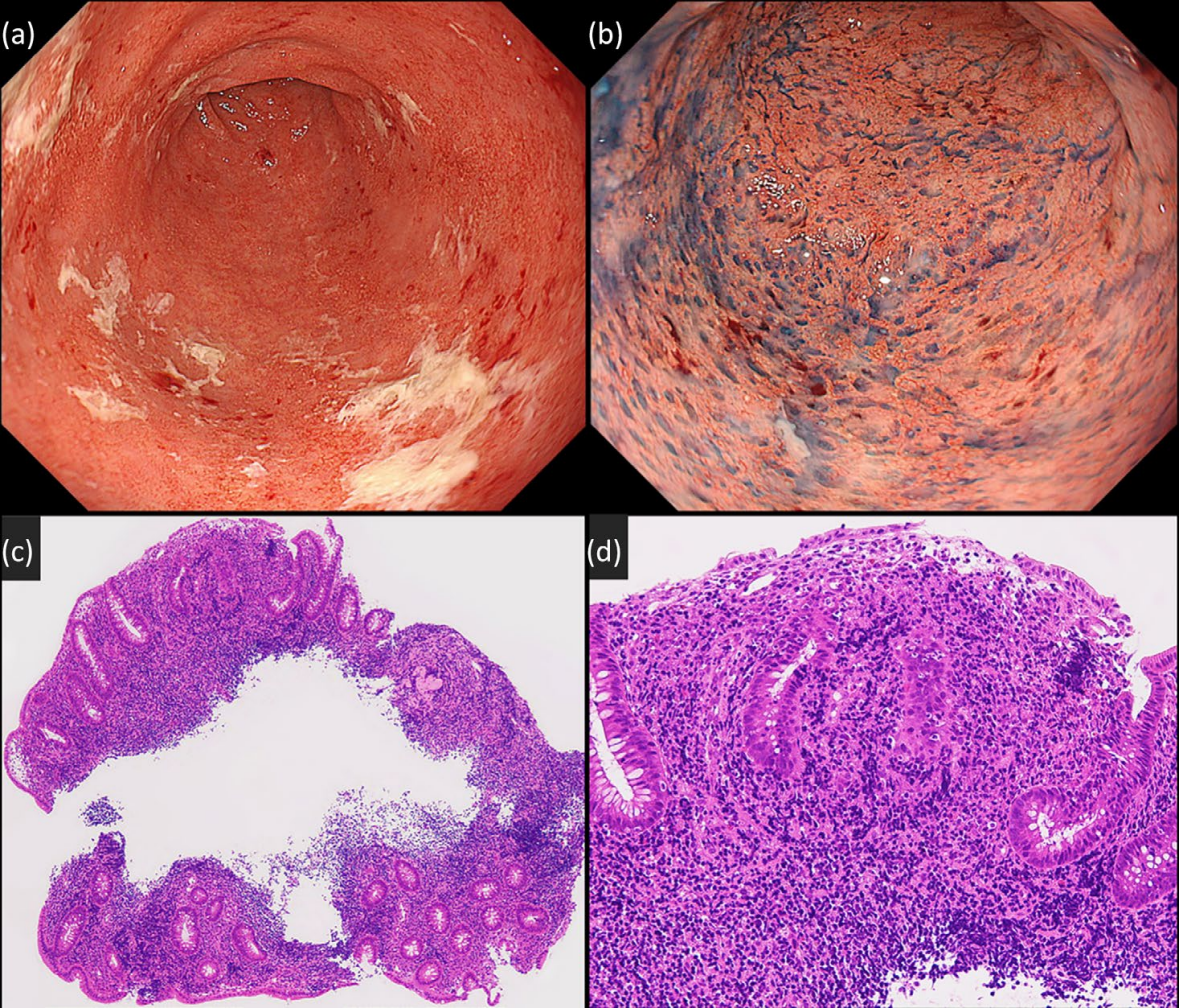
(a) Vaginoscopy reveals continuous inflammation with diffuse erythema, spontaneous bleeding, loss of vascular pattern, and mucosal surfaces coated with purulent mucus in the neovaginal sigmoid colon. (b) Chromoendoscopy with indigo carmine shows mucosal granularity and friability, resembling ulcerative colitis. (c, d) Histological examination of a biopsy specimen stained with hematoxylin and eosin (HE) demonstrates chronic active colitis with crypt distortion, cryptitis, and goblet cell depletion, accompanied by marked infiltration of neutrophils, lymphocytes, and plasma cells (c: 4× objective lens; d: 20× objective lens).

**FIGURE 2:**
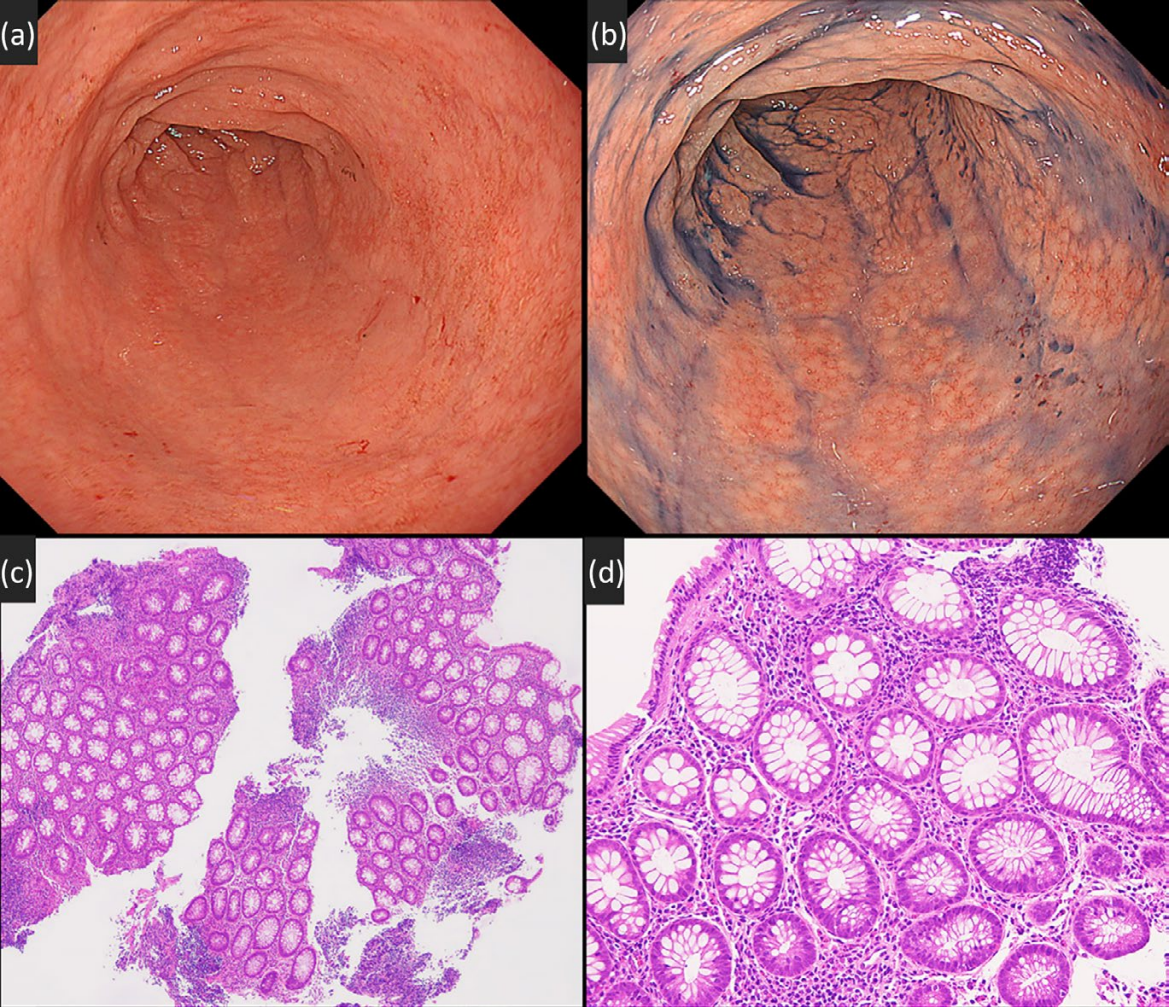
(a) Follow‐up vaginoscopy performed 7 weeks after the initiation of mesalazine treatment reveals near‐complete mucosal normalization. (b) Chromoendoscopy with indigo carmine indicates a trend toward mucosal healing. (c, d) Histological examination of a biopsy specimen stained with hematoxylin and eosin (HE) demonstrates colonic mucosa with no significant abnormalities (c: 4× objective lens; d: 20× objective lens).

## Author Contributions

Shinichiro Kawatoko designed the study, managed the patient's care, performed endoscopic evaluations, reviewed histological specimens, and drafted the manuscript. Marimo Mori and Junji Umeno critically reviewed and revised the manuscript for important intellectual content. All authors have read and approved the final version of the manuscript.

## Funding

The authors have nothing to report.

## Conflicts of Interest

The authors declare no conflicts of interest.

## References

[1] W. B. van der Sluis , M. B. Bouman , N. K. de Boer , B. M. Stubenitsky , D. M. de Leeuw , and A. A. van Bodegraven , “Diversion Neovaginitis After Sigmoid Vaginoplasty: Endoscopic and Clinical Characteristics,” Fertility and Sterility 105 (2016): 738–744. 10.1016/j.fertnstert.2015.11.013 26632208

[2] K. Tominaga , K. Kamimura , K. Takahashi , J. Yokoyama , S. Yamagiwa , and S. Terai , “Diversion Colitis and Pouchitis: A Mini Review,” World Journal of Gastroenterology 24 (2018): 1734–1747. 29713128 10.3748/wjg.v24.i16.1734 PMC5922993

